# Brain connectivity moderated the effects of cognitive intraindividual variability on mobility in cognitively frail older adults

**DOI:** 10.3389/fnagi.2025.1682996

**Published:** 2025-10-31

**Authors:** Jingyi Wu, Jinyu Chen, Juncen Wu, Wayne Lap Sun Chan, Yijian Yang, Chun Liang Hsu

**Affiliations:** ^1^Department of Rehabilitation Sciences, Faculty of Health and Social Sciences, The Hong Kong Polytechnic University, Hong Kong, Hong Kong SAR, China; ^2^Department of Sports Science and Physical Education, The Chinese University of Hong Kong, Hong Kong, Hong Kong SAR, China

**Keywords:** executive function, Timed Up and Go, frailty, cognitive impairment, functional connectivity, fMRI

## Abstract

**Introduction:**

Cognitive frailty, defined by the coexistence of mild cognitive impairment and physical frailty, imposes greater risk of negative health consequences than either condition alone. Cognitive intraindividual variability (IIV), which reflects the extent of fluctuation in cognitive performance, is an early indicator of impaired cognition and mobility. To extend current understanding of the underlying neural mechanisms of increased IIV due to cognitive frailty, this study investigated the association between brain networks, IIV, and mobility.

**Methods:**

A total of 38 community-dwelling cognitively frail/non-cognitively frail older adults (CF and non-CF; *n* = 17 and *n* = 21, respectively) underwent clinical assessments including the Trail Making Test, Stroop Test, Timed Up and Go test (TUG), and resting-state functional magnetic resonance imaging. Dispersion across executive tests was computed to ascertain IIV (IIV-dispersion). Analysis of covariance was used to determine group differences in IIV-dispersion and functional network connectivity adjusted for functional comorbidities. Moderation models were constructed to investigate the role of functional neural networks in the association between IIV-dispersion and TUG performance.

**Results:**

Compared to non-CF group, CF group exhibited greater IIV-dispersion (*p* = 0.042), lower within sensorimotor network (SMN) connectivity, and lower connectivity between the default mode network (DMN), fronto-executive network (FEN), and SMN (all *p* < 0.050). Further, regional DMN-FEN connectivity moderated the relationship between IIV-dispersion and TUG performance (R-sq = 0.427, *p* = 0.001) only among the CF.

**Discussion:**

Greater IIV-dispersion due to cognitive frailty may be underpinned by large-scale altered functional connectivity across networks. However, localized reconfiguration of DMN-FEN connectivity may uniquely represent adaptive compensatory processes by which mobility is protected against the detrimental impact of greater IIV-dispersion secondary to cognitive frailty.

## 1 Introduction

Cognitive frailty is an increasingly prevalent geriatric condition that affects 10% of older adults over 60 years in Asia (Qiu et al., 2022). It is characterized by the co-occurrence of mild cognitive impairment (MCI) with physical frailty as identified by reduced strength, endurance, and diminished physiological function (Kelaiditi et al., 2013). A growing body of evidence demonstrates a bidirectional link between cognitive function and physical frailty in older adults. Specifically, subtle cognitive changes can severely impact functional capacity in those with physical frailty; while physical frailty is a significant predictor of further cognitive decline and poorer functional outcome among cognitively impaired individuals (Buchman et al., 2007; Dubbioso et al., 2023; Gray et al., 2013; Spisto et al., 2025). Consequently, older adults with cognitive frailty are particularly vulnerable to stressors and face significantly elevated risks of falls, disability, dementia progression, and all-cause mortality (Bu et al., 2021).

Findings from the literature established that, while not ubiquitous, executive function deficits are common in older populations with MCI (Gray et al., 2013). Executive functions are higher-order neurocognitive processes that enable individuals to make decisions, engage in purposeful behavior, and plan for the future (Hofmann et al., 2012). The three most extensively studied individual domains of executive function include response inhibition, updating, and set-shifting (Miyake et al., 2000). In particular, response inhibition concerns the ability to suppress the dominant or automatic response; updating focuses on the replacement of old memory with new information; and set-shifting refers to one’s capacity to switch between various mental tasks.

Previous studies have also demonstrated a notable association between physical frailty and executive function in older adults. For example, a longitudinal study of 29,591 older adults observed a significant relationship between physical frailty and executive function over a three-year follow-up period (Courish et al., 2025). Similarly, faster decline in executive function, particularly set-shifting, predicted the onset of physical frailty over nine years in older adults initially free of dementia and frailty (Gross et al., 2016). Moreover, individuals with cognitive frailty exhibit greater impairments in executive function domains such as set-shifting and updating compared to those without cognitive frailty (Delrieu et al., 2016).

Cognitive assessments, which included but are not limited to executive function, are commonly evaluated as overall scores (i.e., mean scores). However, overall scores only present one aspect of an individual’s cognitive capabilities (i.e., mean-level cognitive performance), whereas intraindividual variability (IIV) is another important facet of cognition that reflect how well one can maintain stable level of cognitive performance despite the perturbations from multiple cognitive stimuli (Hultsch et al., 2008; Robert et al., 2015; Susan Vandermorris, 2015). Broadly, IIV can be categorized into three types: (1) inconsistency, referring to trial-to-trial fluctuations within a single task during one testing session, capturing moment-to-moment variability (Hultsch et al., 2002); (2) variability, reflecting changes in performance on a single task across multiple testing occasions, capturing longer-term fluctuations (Hultsch et al., 2000; Nesselroade and Salthouse, 2004; Stuss et al., 2003); and (3) dispersion, reflecting variability across multiple cognitive tests within a single testing occasion. Dispersion can be further divided into within-domain dispersion (variation across tests within the same cognitive domain) (Mulet-Pons et al., 2023b; Scott et al., 2023; Stuss et al., 2003) and across-domain dispersion (variation across tests spanning multiple cognitive domains) (Halliday et al., 2018; Hilborn et al., 2009; Holtzer et al., 2008; Mulet-Pons et al., 2023b; Scott et al., 2023).

Most studies quantify IIV using the intraindividual standard deviation of performance scores on each cognitive test or domain, which indexes the variability or spread of an individual’s performance around their own mean score (Hilborn et al., 2009; Mulet-Pons et al., 2023a; Scott et al., 2023). Increased standard deviation indicates greater IIV and is often associated with poorer mean performance. Importantly, this suggests that the interpretation of IIV is not dependent on which cognitive domains are included in the calculation; rather, differences in cognitive domains primarily reflect the magnitude of perturbation or cognitive challenge presented by the tasks. Given the relevance of executive function to MCI and physical frailty, the present study focuses on IIV-dispersion within executive function. This approach provides a comprehensive view of cognitive function and insights into how executive processes interact with overall cognitive health. It is particularly relevant for older adults with cognitive frailty, who often exhibit impairments across multiple executive function domains rather than isolated deficits on single tests (Delrieu et al., 2016; Kelaiditi et al., 2013).

Studies suggest IIV-dispersion may be a more sensitive indicator for aging-related cognitive deficits than standard evaluation based on mean score (Hilborn et al., 2009; Holtzer et al., 2008; Lindenberger and Baltes, 1997; Wu et al., 2025). For instance, a previous study showed notable differences in IIV-dispersion between young-old and old-older groups, but no differences were observed in cognitive performances assessed as total scores (Lindenberger and Baltes, 1997). In addition, using IIV-dispersion, researchers were able to effectively discriminate between those with and without cognitive decline (Hilborn et al., 2009), as well as predict the incidence of dementia (Holtzer et al., 2008). Of note, our previous work found that IIV significantly differentiated the physically frail older adults from those without physical frailty (Wu et al., 2025). In addition to being a sensitive indicator for cognitive deficits, recent study also shed light on the link between intraindividual variability and mobility. Specifically, higher IIV in simple reaction time of Stroop task (i.e., IIV-inconsistency) was significantly associated with poorer mobility, as measured by the Timed Up and Go (TUG) test (Dimri et al., 2024). Notably, baseline TUG performance also predicted IIV after 6 months (Dimri et al., 2024), which illustrated the well-established bidirectional relationship between aging-related cognitive and mobility decline (Dimri et al., 2024).

As the brain is central to both cognitive function and mobility, evidence from neuroimaging studies suggests that executive function and mobility share numerous overlapping functional brain networks (Hsu et al., 2019; Jor’dan et al., 2017; Poole et al., 2018). Specifically, the default mode network (DMN) is involved in processes for integrating past experiences to plan for future actions (Andrews-Hanna et al., 2007; Buckner et al., 2008). The fronto-executive network (FEN) is primarily involved in executive functions, monitoring errors in top-down control, and sustaining an extended, task-dependent cognitive state (Dosenbach et al., 2006; Seeley et al., 2007). The sensorimotor network (SMN) contributes to motor planning, initiation, execution, and coordination (Solodkin et al., 2004). Importantly, greater IIV-dispersion is correlated with disruptions in these networks. For example, in healthy middle-aged adults, study found greater IIV-dispersion across cognitive domains (i.e., processing speed, executive function, and episodic memory) was associated with lower resting-state functional connectivity within the DMN and between DMN and dorsal attention network, which comprised of partially overlapping regions with the FEN examined in our study (Mulet-Pons et al., 2023a). Additionally, greater IIV-dispersion within the domain of processing speed, as assessed by two tests, was associated with lower DMN connectivity (Mulet-Pons et al., 2023a). In a sample of 63 cognitively unimpaired middle-aged and older adults, researchers reported that greater IIV-dispersion across cognitive domains was correlated with lower functional connectivity within the DMN and SMN (Lin and McDonough, 2022). These findings suggest that the DMN may be related to IIV-dispersion both within and across cognitive domains.

Within these networks, key hubs such as the bilateral middle temporal gyrus (BMTG; DMN) and bilateral inferior frontal gyrus (BIFG; FEN) play crucial roles in cognitive processes and mobility. For instance, in older adults, executive function (i.e., response inhibition and set-shifting) is significantly linked to connectivity of the BIFG (Gogniat et al., 2022), and walking performance is positively associated with activity of the right inferior frontal gyrus (Demnitz et al., 2017). Similarly, studies indicated that the disrupted connectivity of the middle temporal gyrus is linked to impaired executive function (i.e., set-shifting) and early-stage Alzheimer’s disease (Buckner et al., 2008; Oosterman et al., 2012). Functional magnetic resonance imaging (fMRI) studies have also demonstrated greater bilateral MTG activity during imagined standing and walking tasks in older adults (Zwergal et al., 2012). Adaptive functional network reorganization may mitigate cognitive impairment in older adults. For instance, compared with cognitively impaired older adults with lower connectivity within DMN and FEN, those with greater connectivity within these networks showed better executive function under increased white matter lesion load (Gu et al., 2024). Likewise, functional connectivity between DMN (i.e., medial frontal cortex) and FEN (i.e., anterior cingulate cortex) moderated the negative impact of white matter lesion on executive function in older adults (Benson et al., 2018). Moreover, through moderation analyses, study reported that compared with cognitively unimpaired older adults with lower connectivity within executive network, those exhibiting greater connectivity within executive network showed better mobility and balance, as assessed by expanded short physical performance battery, in face of higher Amyloid beta load (Laurienti et al., 2025). However, the complex interplay between IIV-dispersion, mobility, and brain function remained unexplored.

Therefore, this cross-sectional study aimed to examine the association between IIV-dispersion, mobility, and functional connectivity in older adults with and without cognitive frailty. We specifically focused on internetwork connectivity between anatomical regions found within three functional networks (i.e., DMN, FEN, and SMN) due to their biological relevance to cognitive intraindividual variability and mobility. We hypothesized that compared with non-cognitively frail older adults, older adults with cognitive frailty would exhibit aberrant intra- and inter-network functional connectivity of the DMN, FEN, and SMN. We also hypothesized that inter-network connectivity of the DMN and FEN (i.e., BMTG-BIFG) may moderate the relationship between higher IIV-dispersion and poorer mobility.

## 2 Materials and methods

### 2.1 Recruitment

This cross-sectional study comprised of a total of 38 community-dwelling older adults between the age of 65–90 years with and without cognitive frailty as identified by presence of both probable MCI and physical frailty. Participants were recruited from local community centers and non-government organizations between September 2023 and April 2024. Ethical approval was obtained from the Institutional Review Board of the Hong Kong Polytechnic University (HSEARS20230131001). Written informed consent was obtained for all study participants enrolled in the study.

### 2.2 Cognitive frailty characterization

According to the IANA/IAGG consensus in published in 2013, cognitive frailty is a conditioned defined as having physical frailty and cognitive impairment, but without diagnosis of AD or other dementia (Kelaiditi et al., 2013).

Within the context of this study, we defined cognitive frailty as those with probable MCI and physical frailty. Specifically, probable MCI was assessed using the Montreal Cognitive Assessment (MoCA) and physical frailty was assessed using the Short Physical Performance Battery (SPPB), as recommended by the European Medicines Agency, due to its reliability, validity, and ease of use in clinical settings (Agency, 2018). Probable MCI was assessed by the Hong Kong version of the Montreal Cognitive Assessment (MoCA) (Yeung et al., 2020). The MoCA is comprised of seven domain-specific components (visual-spatial, naming, attention, language, abstraction, delay, and orientation). An additional point was given to participants who received ≤ 12 years of education (Nasreddine et al., 2005). The total score ranges from 0 to 30 points, with a score ≥ 26/30 indicating unimpaired global cognition (Nasreddine et al., 2005), 18–25/30 indicating probable MCI, and < 18/30 indicating signs of dementia (Yeung et al., 2020). The SPPB is a validated instrument that has good accuracy in detecting sarcopenia and frailty in the community setting. Study showed that SPPB of ≤ 9 points is sensitive and specific for identifying physical frailty (da Câmara et al., 2013; Ramírez-Vélez et al., 2021). The test consists of three subscales (standing balance test, 4-meter walk at usual pace, and timed chair sit-to-stand test). Each subscale is scored with a maximum of four points for a total of 12 points, with a higher score indicating better general mobility.

Participants were identified as cognitively frail with a score of ≤ 9/12 on the SPPB and 18–25/30 on the MoCA. Non-cognitively frail older adults were identified with a score of > 9/12 on the SPPB and ≥ 26/30 on the MoCA.

### 2.3 Inclusion criteria

Participants were included if they: (1) were between 65 and 90 years old; (2) lived in the community; (3) were able to ambulate up to four meters with or without assistive devices; (4) were able to provide written informed consent by his/her own behalf; and (5) can understand verbal and written Cantonese and/or English.

### 2.4 Exclusion criteria

Participants were excluded if they: (1) had magnetic resonance imaging (MRI) contraindications; (2) were diagnosed with neurodegenerative conditions (i.e., dementia, Parkinson’s, Alzheimer’s disease, Amyotrophic lateral sclerosis, and stroke); (3) lived in nursing home or other care facilities/institutions; and (4) were taking fixed dose of medication or had been taking medications known to potentially affect cognitive and physical function (e.g., psychotropic medications), as identified through a review of their medication lists recorded in the health software. Medications were categorized based on their properties (e.g., antipsychotics, antidepressants etc.) to inform eligibility decisions.

Self-reported diagnosis of MCI was not part of the inclusion/exclusion criteria.

### 2.5 Outcome measures

Participant demographic baseline data included age, sex, height, weight, body mass index, waist-to-hip ratio, and years of education. Clinical information comprised depression, assessed using the 15-item Geriatric Depression Scale (GDS), a validated diagnostic screening tool for elderly people, and physical fatigue, measured with the Fatigue Severity Scale (FSS). A higher score on these scales indicates a more severe condition. Sleep duration was determined based on participants’ self-reported sleep over the past 24 h and the previous seven days. Physical activity levels were evaluated using the Physical Activity Scale for the Elderly (PASE), where a higher score indicates one was more physically active over the past week (Ngai et al., 2012).

#### 2.5.1 Mobility

TUG test is a validated measure of gait and mobility (Shumway-Cook et al., 2000). The test required participants to stand up from a standard chair without armrest, walk 3 meters, cross a line marked on the floor, turn around, walk back to the chair, and sit down while being timed throughout the examination. The TUG test was performed twice, and the average time taken to perform the test was calculated.

#### 2.5.2 Executive function

Set-shifting and response inhibition were assessed using the Trail Making Test and the Stroop test, respectively.

In the Trail Making Test (Part A & B) (Spreen and Strauss, 1998), participants were asked to connect circled numbers sequentially (Part A) or alternate between numbers and letters sequentially (Part B). A standard score is derived by calculating the difference between Part B and Part A completion times (B-A), with lower scores indicating better set-shifting ability.

In the Stroop Test (Part I, II, and III) (Graf et al., 1995), participants initially read out the color of printed words (like “BLUE”). Then, they named the colors of “X”s presented in various hues. Finally, they were shown a page with color words printed in incongruent colored inks (e.g., the word “BLUE” printed in red ink), where they were required to name the ink color, disregarding the word’s meaning. We timed their performance in each phase and determined the difference between the last and second tasks’ completion times. A better ability to inhibit responses is indicated by shorter intervals between the two parts (Stroop-interference = Part III—Part II) (Shum et al., 1990).

#### 2.5.3 Computation of intraindividual variability (IIV)

Recent evidence has highlighted notable association between TUG performance and executive functions (i.e., set-shifting and response inhibition) in older adults. For instance, a cross-sectional study reported a moderate correlation between TUG performance and set-shifting ability, as assessed by the Trail Making Test Part B minus Part A, while no significant relationship was observed with other executive function measures such as phonemic and semantic fluency in older adults with and without probable MCI (Falck et al., 2017). Similarly, in a sample of 201 older adults with probable MCI, poorer TUG performance was found to be associated with greater Stroop interference and longer completion times on the Trail Making Test Part B, further underscoring the link between TUG deficits and specific executive function impairments (McGough et al., 2011). Additionally, in accordance with previous studies that computed IIV-dispersion, we treated each cognitive subtest as an independent measure (Bangen et al., 2019; Hilborn et al., 2009; Koscik et al., 2016). This was based on the rationale that each component of the tests captures a correlated but separate cognitive domain (e.g., Stroop I–processing speed; Stroop II–processing speed and language processing; Stroop III -conflict monitoring/resolution and response inhibition). Under this framework, it is reasonable to consider that our IIV-dispersion calculation was based on four cognitive measures (i.e., Trail Making Test Part A, Trail Making Test Part B, Stroop II, and Stroop III).

Computation of IIV-dispersion was performed through four steps, based on the methodology described by Holtzer et al. (2008), which similarly employed within-sample standardization for IIV calculation (Holtzer et al., 2008). First, the raw score in each cognitive test (i.e., Trail Making Test B-A and Stroop-interference) was z-transformed separately according to the distribution of entire older adults [Equation (1)]. Second, the sum of each participant’s z-transformed score for each of the two aspects of executive function was calculated by Equation (2) (Holtzer et al., 2008). Third, the variability in each of the two aspects of executive function was calculated by Equation (3) (Holtzer et al., 2008). Finally, the square root of the sum of variability in two aspects of the executive function was calculated by Equation (4) to derive the amount of dispersion across two aspects of the executive function.


(1) $$Z_{i\,k}=\frac{X-\mu }{\sigma }$$

(2) $$A_{i}=\sum_{k=1}^{K}Z_{i\,k}$$

(3) $$V\,a\,r\,i\,a\,b\,i\,l\,i\,t\,y=\sqrt{\frac{{\left(Z_{i\,k}-A_{i}\right)}^{2}}{\left(k-1\right)}}$$

(4) $$\mathrm{IIV}=\sqrt{\sum_{k=1}^{K}\frac{{\left(Z_{\mathrm{ik}}-A_{i}\right)}^{2}}{\left(k-1\right)}}$$

Z_ik_ was the kth executive test score for the ith individual. μ was the mean value of all tests. X was the raw score of each test. σ represented the standard deviation of all tests. K represented the number of cognitive tests. A_i_ was the individual’s sum Z transformed score based on the number of tests.

#### 2.5.4 Covariates

Total number of comorbid conditions was assessed through the Functional Comorbidity Index (FCI), with a maximum score of 18 (Groll et al., 2005). FCI includes a broad range of chronic conditions that affect functional status, specifically: arthritis, osteoporosis, asthma, chronic obstructive pulmonary disease, acquired respiratory distress syndrome, emphysema, angina, congestive heart failure, heart attack, neurological diseases such as multiple sclerosis or Parkinson’s disease, stroke or transient ischemic attack, peripheral vascular disease, diabetes, upper gastrointestinal conditions, depression, anxiety or panic disorders, visual impairments, severe hearing impairment, degenerative disc disease, and obesity defined as a body mass index greater than 30. A lower score indicates fewer comorbidities.

Two assessors evaluated all the participants. The clinical assessment was conducted in the university-based rehabilitation laboratory and lasted approximately one and a half hours.

### 2.6 MRI acquisition

MRI sessions were conducted within 7 days of the clinical assessments at the University Research Facility in Behavioral and Systems Neuroscience of the Hong Kong Polytechnic University using the research dedicated 3T Siemens Prisma scanner with 32-channel head coil. High resolution structural image was collected with one three-dimensional 1 mm isotropic T1w MPRAGE (TR = 2,530 ms, TE = 3.04 ms, TI = 800 ms, flip angle = 10°, FOV = 256 mm × 256 mm × 220 mm). Resting-state functional image was collected with one 1 mm isotropic T2w (SPACE) image (TR = 4,000 ms, TE = 406 ms, flip angle = 90°, FOV = 260 mm × 228 mm × 176 mm).

#### 2.6.1 Functional MRI data processing

Functional network connectivity was quantified with resting state fMRI data. Participants were instructed to rest with eyes open for approximately 12 min. After removal of the first four volumes to allow the signal to reach a steady state, the resting state fMRI data were preprocessed using rigid body motion correction with MCFLIRT. We used a standard of less than 2 mm in absolute displacement and less than 0.5 mm in relative displacement as cut-offs for motion threshold. Based on this, no participants were excluded from the analysis. Spatial smoothing was used via a 6.0 mm Full-Width-Half-Maximum Gaussian kernel, high-pass temporal filtering was performed to exclude confounding signals from frequencies below 0.008 Hz. Spikes in signals due to motion were first removed from the time-series data through FSL’s motion outlier tool followed by an Independent Component Analysis based Automatic Removal of Motion Artifacts to remove motion-related artifacts. Nuisance signals from cerebral spinal fluid and white matter were regressed out via general linear model.

#### 2.6.2 Functional connectivity analysis

Regions of interest (ROIs) within the DMN, FEN, and SMN were selected *a priori* based on coordinates from a prior study (see Supplementary Table 1 for full details) (Papanicolaou, 2017). The DMN included the posterior cingulate cortex (PCC), ventral and superior frontal medial cortices (FMC), MTG, para-hippocampal gyrus (PHG), middle frontal gyrus (MFG), and lateral occipital cortex (LOC) (Cabeza et al., 2017). The FEN included the anterior lateral prefrontal cortex (RALPFC), insular sulcus (INS), prefrontal cortex (PFC), IFG, and anterior cingulate gyrus (CING) (Cabeza et al., 2017). The SMN included the primary motor cortex (PCG), cerebellum (CB), premotor area (PM), and supplementary motor area (SMA) (Wu et al., 2009). For each ROI, preprocessed time-series data were extracted with 14 mm spherical regions of interest drawn around their respective MNI coordinates in standard space. ROIs time-series data were subsequently cross-correlated to establish functional connectivity maps of their associated neural networks, in which pairwise correlation between time-series extracted from ROI listed above was calculated. Correlation estimates were then Fisher’s z transformed to improve normality before subsequent statistical analyses.

### 2.7 Statistical analysis

R software v.4.3.2 was used to perform all statistical analyses (R Development Core Team, 2014). Independent *t*-tests, Mann–Whitney U tests, and chi-squared tests (for ratio and nominal data, respectively) were performed to compare the differences in demographic and clinical measurements between the two groups (i.e., CF group and non-CF group). Pearson or Spearman correlation analyses were performed to examine the relationship among IIV-dispersion, sleep duration, and PASE in each group based on the normality of the distribution of each variable. Boxplot method and standard deviations (i.e., 3SD away from the mean) were carried to detect the outlier related to primary outcome measurements [i.e., Trail Making Test B–A and Stroop interference (III–II)]. We conducted two Multivariate Analysis of Covariance (MANCOVAs), one for each variable type (i.e., TUG performance, and executive function, as well as functional connectivity), adjusting for FCI scores. Then individual ANCOVAs was used to determine whether there are significant differences between the two groups in TUG performance, IIV-dispersion, and intra- and inter-network functional connectivity (DMN, SMN, DMN-FEN, DMN-SMN, FEN-SMN), adjusting for FCI scores. The adjustment was applied by weighting the difference between each group’s average covariate value and the grand mean of the covariate by the model’s common regression slope. ANCOVA was performed using the Anova() function from the car package. Adjusted means were estimated marginal means using the lsmeans package (R version 4.3.2). Corrections for multiple comparison was performed using Bonferroni. The effect size of the difference between groups was calculated using partial eta squared, where values of 0.01, 0.06, and 0.14 corresponded to small, medium, and large effect sizes, respectively. Moderation analyses were conducted using the PROCESS macro. Assumptions of linear regression, including linearity and homoscedasticity were verified and met (see Supplementary Figure 1 for full details). To ensure our results were not influenced by multicollinearity, IIV- dispersion and moderator were mean-centered prior to creating the interaction term (see Supplementary Tables 2, 3 for full details). Regions of interest highlighting DMN-FEN connectivity were selected as moderators given that these particular brain areas (i.e., frontal and temporal cortex) were established to be significantly related to executive function (Voss et al., 2010). Therefore, we constructed two separate models (one for the non-CF group and one for the CF group) to test the direct effect of IIV-dispersion on the TUG performance as well as to investigate the moderation effect of functional connectivity between DMN and FEN on the association between IIV-dispersion and the TUG performance (see Supplementary Figure 2 for full details). To further understand the nature of this interaction, the conditional effect of IIV-dispersion (simple slopes) on TUG performance was estimated at three levels of the values of the moderators [i.e., functional connectivity between DMN and FEN: low (i.e., mean–SD), middle (i.e., mean), high (i.e., mean+SD)]. The statistical significance level was set at *p* < 0.05 for all tests.

## 3 Results

### 3.1 Participants

**FIGURE 1:**
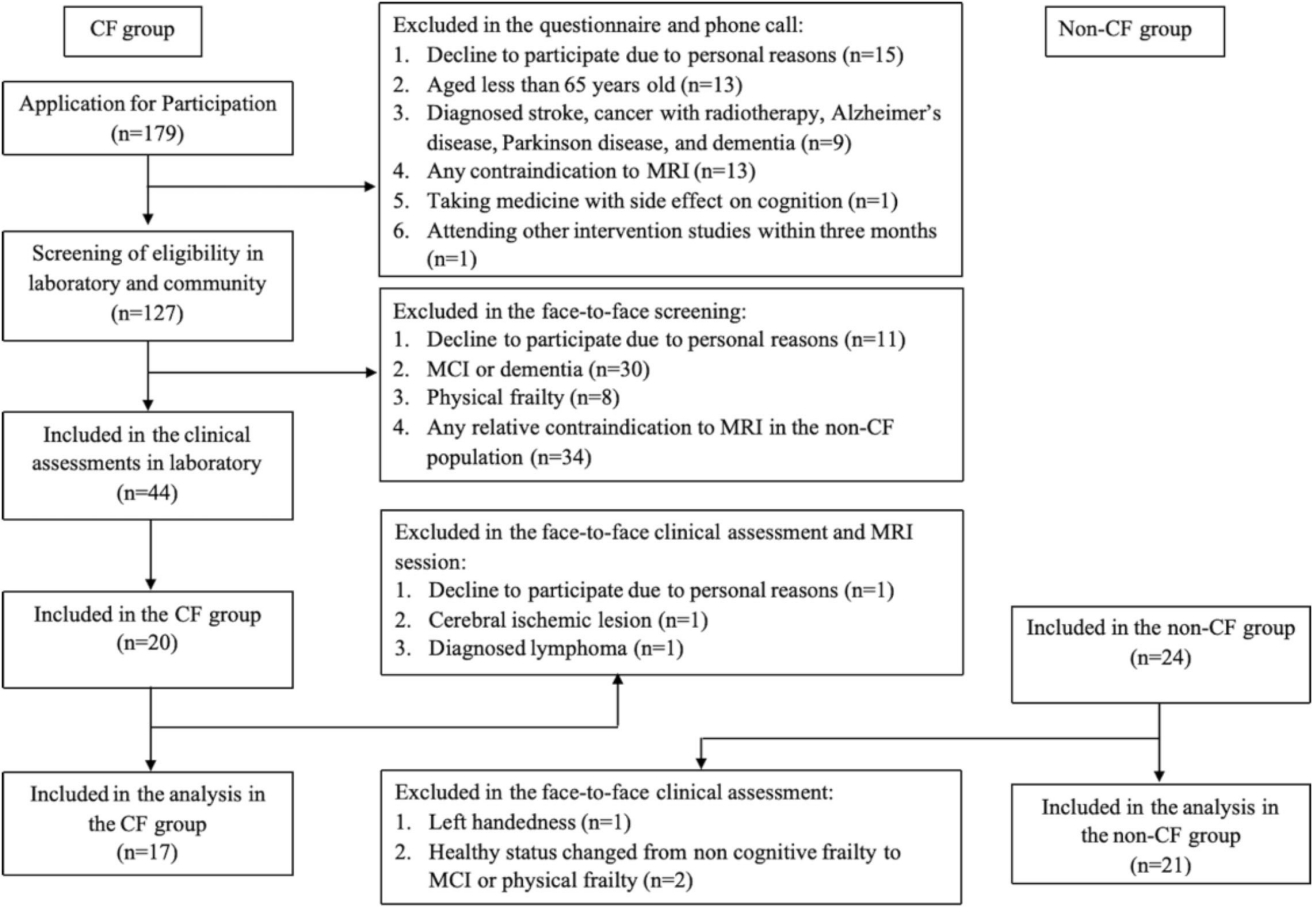
Study recruitment flowchart.

Forty-four participants were enrolled, and thirty-eight participants [i.e., CF group (*n* = 17) and non-CF group (*n* = 21)] were analyzed (Figure 1). After removing one outlier (i.e., Stroop interference) from the non-CF group, the non-CF group included 20 older adults. Participant characteristics are detailed in Table 1. Compared to the non-CF group, the CF group on average slept less, were less physically active, measured by the PASE, and had more comorbidities, assessed by FCI. No other differences in characteristics were observed between the groups (*p* > 0.05) (Table 1).

**TABLE 1:** Study participant characteristics.

Demographic data	CF group (*n* = 17) Mean (SD)	Non-CF group (*n* = 20) Mean (SD)	*P*-value
Age (years)	74.059 (3.030)	73.050 (2.911)	0.152
Sex (M/F)	5/12	9/11	0.530
Height (cm)	158.084 (8.685)	161.858 (6.966)	0.160
Weight (kg)	59.413 (10.604)	61.006 (10.971)	0.657
BMI	23.732 (3.471)	23.157 (2.945)	0.595
WtoH	0.901 (0.051)	0.886 (0.089)	0.541
Education (years)	10.471 (5.387)	13.600 (3.409)	0.060
GDS	3.000 (2.761)	1.800 (2.238)	0.110
FSS	36.176 (13.520)	33.550 (11.033)	0.527
Sleep duration (last 24 h)	5.765 (1.897)	7.125 (1.223)	0.017
Sleep duration (last 7 days)	5.735 (1.542)	7.150 (1.377)	0.008
PASE	115.351 (43.870)	157.495 (57.584)	0.016
FCI	1.647 (0.996)	1.000 (0.858)	0.042
MoCA	22.941 (1.983)	27.850 (1.182)	–
SPPB	7.765 (1.300)	11.400 (0.681)	–

CF, cognitively frail older adults; SD, standard deviation; M, male; F, female; BMI, body mass index; WtoH, waist to hip ratio; GDS, Geriatric Depression Scale; FSS, Fatigue Severity Scale; PASE, Physical Activity Scale for the Elderly; FCI, Functional Comorbidity Index; MoCA, Montreal Cognitive Assessment; SPPB, Short Physical Performance Battery.

### 3.2 Group differences in TUG performance and executive function

After adjusting for FCI, participants in the CF group showed worse TUG performance with a large effect size (*p* < 0.001, η^2^ = 0.461) and greater IIV-dispersion with a medium effect size (*p* = 0.042, η^2^ = 0.116), compared with the non-CF group (Table 2) (see Supplementary Table 4 for full details following adjustment for FCI and years of education).

**TABLE 2:** Participant mobility, executive functions, and network functional connectivity.

Variables	Mean (SD)		Adjusted Mean (SD)		Effect Size (η^2^)	*P*-value
	CF group (*n* = 17)	Non-CF group (*n* = 20)	CF group (*n* = 17)	Non-CF group (*n* = 20)		
**Mobility measure**						
TUG	11.349 (1.802)	8.216 (1.545)	11.381 (1.748)	8.189 (1.740)	0.461	0.000
**Executive function measure**						
Trail Making Test A	45.404 (20.578)	32.324 (9.724)	46.099 (16.307)	31.732 (16.229)	0.166	0.014
Trail Making Test B	152.341 (75.390)	80.977 (24.643)	156.552 (55.567)	77.398 (55.293)	0.342	0.000
Trail Making Test B-A	106.936 (65.619)	48.654 (19.199)	110.452 (48.343)	45.665 (48.075)	0.319	0.000
Stroop I	49.145 (10.887)	40.801 (6.225)	49.405 (9.071)	40.581 (9.029)	0.195	0.007
Stroop II	63.905 (14.711)	52.753 (7.420)	63.856 (11.908)	52.795 (11.851)	0.181	0.010
Stroop III	122.764 (30.514)	95.014 (21.641)	123.495 (27.287)	94.393 (27.150)	0.226	0.003
Stroop interference	58.858 (24.256)	42.262 (18.087)	59.639 (22.067)	41.598 (21.958)	0.146	0.021
IIV-dispersion	1.523 (0.821)	0.943 (0.459)	1.467 (0.664)	0.991 (0.662)	0.116	0.042
**Functional connectivity**						
**SMN**						
RPCG-RCB	0.395 (0.339)	0.78 (0.347)	0.405 (0.363)	0.772 (0.358)	0.251	0.005
**DMN-FEN**						
BMTG-BIFG	0.105 (0.441)	0.401 (0.320)	0.107 (0.400)	0.399 (0.398)	0.120	0.038
BMTG-RALPFC	−0.006 (0.292)	0.216 (0.359)	−0.024 (0.342)	0.232 (0.340)	0.125	0.035
**DMN-SMN**						
BLOC-LCB	0.056 (0.319)	0.349 (0.331)	0.070 (0.338)	0.337 (0.340)	0.136	0.027
BLOC-LPCG	−0.056 (0.354)	0.235 (0.349)	−0.080 (0.363)	0.256 (0.362)	0.181	0.010
BLOC-RCB	−0.041 (0.299)	0.292 (0.309)	−0.021 (0.313)	0.274 (0.313)	0.185	0.009
BLOC-RPCG	−0.290 (0.392)	0.042 (0.386)	−0.295 (0.408)	0.047 (0.407)	0.153	0.018
FMC-SMA	−0.538 (0.466)	−0.200 (0.386)	−0.542 (0.445)	−0.196 (0.443)	0.134	0.028

Covariate was evaluated at FCI = 1.30; SD, standard deviation; CF, cognitively frail older adults; TUG, timed-up-and-go test; SMN, sensorimotor network; RPCG, right precentral gyrus; RCB, right cerebellum; DMN, default mode network; FEN, fronto-executive network; BMTG, bilateral middle temporal gyrus; BIFG, bilateral inferior frontal gyrus; RALPFC, right anterior lateral prefrontal cortex; LCB, left cerebellum; LPCG, left precentral gyrus; FMC, frontal medial cortex; SMA, supplementary motor area. For brevity, only significant differences were reported with Bonferroni correction.

### 3.3 Group differences in functional connectivity

**FIGURE 2:**
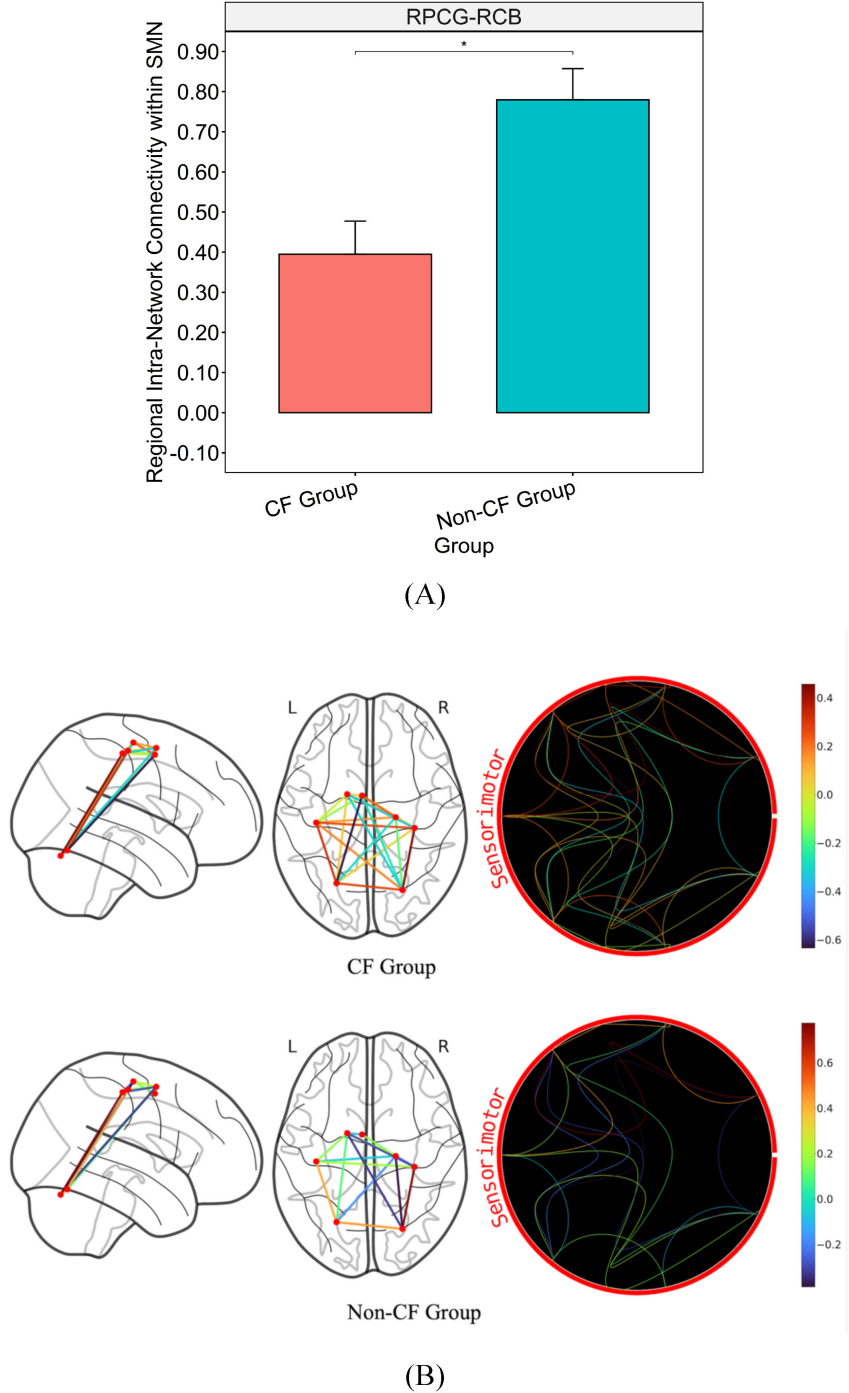
Bar graph illustrating the mean and standard error of group differences in within SMN connectivity. **(A)** Graph on the top illustrates the differences in connectivity strength of major hubs from within the SMN (*p* < 0.05). **(B)** Brain map on the bottom illustrates disparities in connectivity strength between regions of within the SMN. *Significance between-group at *p* < 0.05. Intra-network connectivity is reflected via edges as well as colored heat map (warmer colors reflect positive connectivity; cooler colors reflect negative connectivity) estimated based on calculated connectivity coefficient threshold at 1.7 < Z < 3.1.

After adjusting for FCI, the CF group showed lower connectivity than the non-CF group within the SMN (Figures 2A, B). Particularly between the right primary motor cortex (RPCG) and the right cerebellum (RCB) of the SMN with a large effect size (*p* = 0.005, η^2^ = 0.251; Table 2 and Figures 2A, B).

**FIGURE 3:**
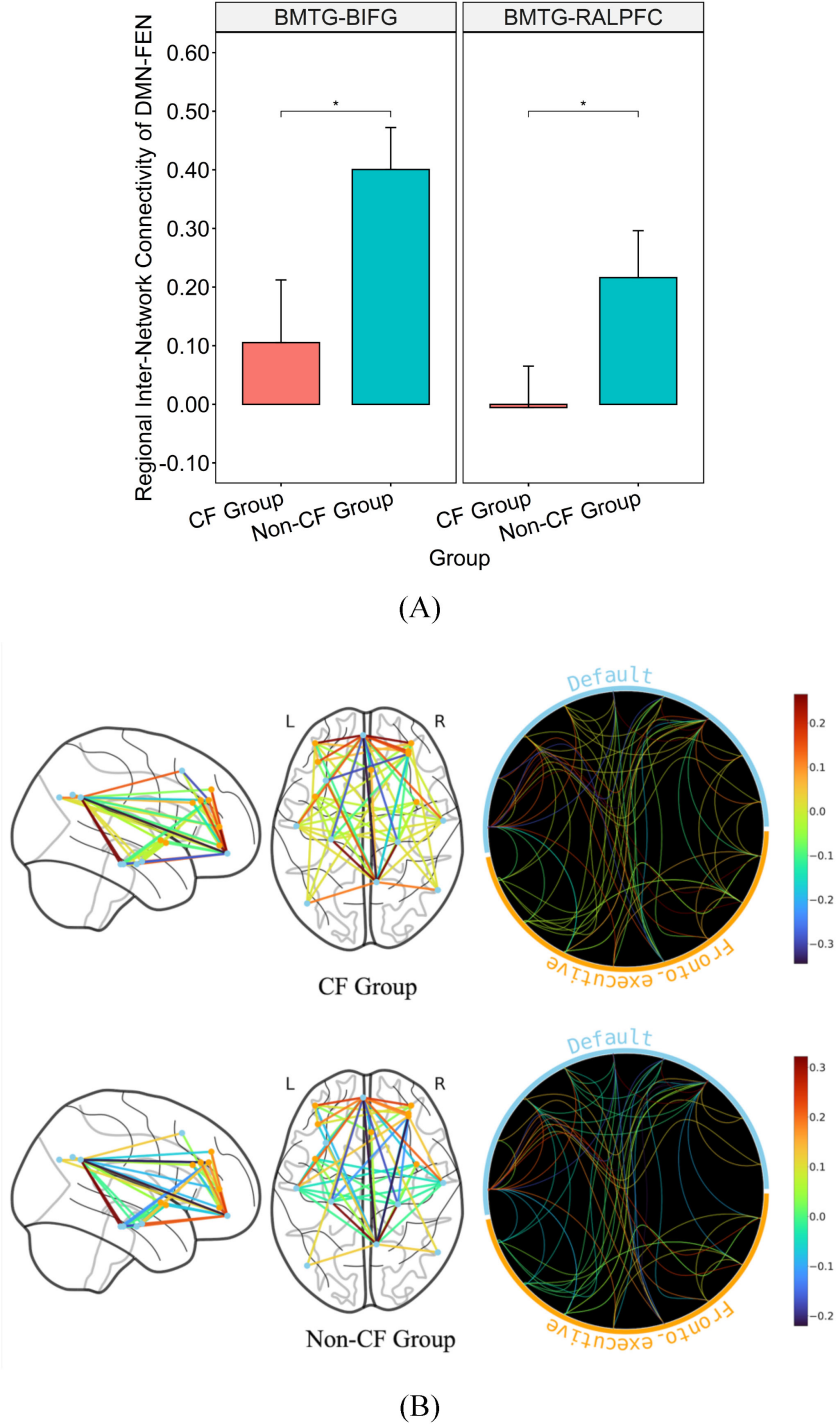
Bar graph illustrating the mean and standard error of group differences in DMN-FEN connectivity. **(A)** Graph on the top illustrates the differences in connectivity strength of major hubs from the DMN and FEN (*p* < 0.05). **(B)** Brain map on the bottom illustrates disparities in connectivity strength between regions of the DMN and FEN. *Significance between-group at *p* < 0.05. Inter-network connectivity is reflected via edges as well as colored heat map (warmer colors reflect positive connectivity; cooler colors reflect negative connectivity) estimated based on calculated connectivity coefficient threshold at 1.7 < Z < 3.1.

**FIGURE 4:**
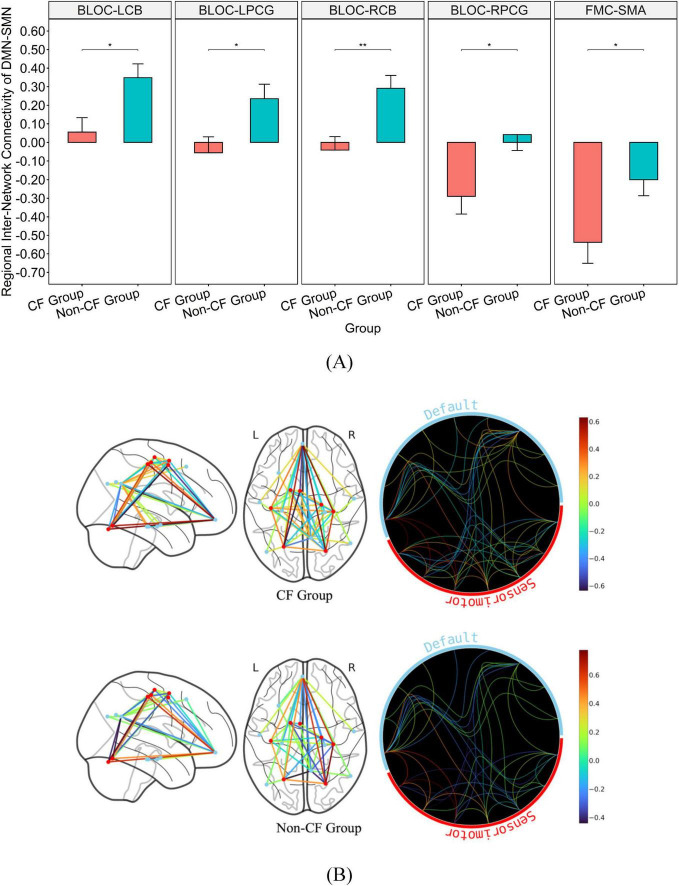
Bar graph illustrating the mean and standard error of group differences in DMN-SMN connectivity. **(A)** Graph on the top illustrates the differences in connectivity strength of major hubs from the DMN-SMN (*p* < 0.05). **(B)** Brain map on the bottom illustrates disparities in connectivity strength between regions of the DMN and SMN. *Significance between-group at *p* < 0.05. **Significance between-group at *p* < 0.01. Inter-network connectivity is reflected via edges as well as colored heat map (warmer colors reflect positive connectivity; cooler colors reflect negative connectivity) estimated based on calculated connectivity coefficient threshold at 1.7 < Z < 3.1.

Additionally, the non-CF group exhibited significantly greater regional connectivity than the CF group between DMN and FEN with a medium effect size (BMTG-BIFG, *p* = 0.038, η^2^ = 0.120; BMTG-RALPFC, *p* = 0.035, η^2^ = 0.125; Table 2 and Figures 3A, B). We also found that participants in the CF group demonstrated notably lower overall connectivity (BLOC-LCB, *p* = 0.027, η^2^ = 0.136; BLOC-LPCG, *p* = 0.010, η^2^ = 0.181; BLOC-RCB, *p* = 0.009, η^2^ = 0.185; BLOC-RPCG, *p* = 0.018, η^2^ = 0.153; Table 2 and Figures 4A, B) as well as greater regional anti-connectivity between the DMN and SMN (FMC-SMA, *p* = 0.028, η^2^ = 0.134; Table 2 and Figures 4A, B) when compared to the non-CF group.

No significant group differences were detected in functional connectivity within DMN or between FEN and SMN (*p* > 0.05).

### 3.4 Moderation model on the DMN and FEN connectivity

**FIGURE 5:**
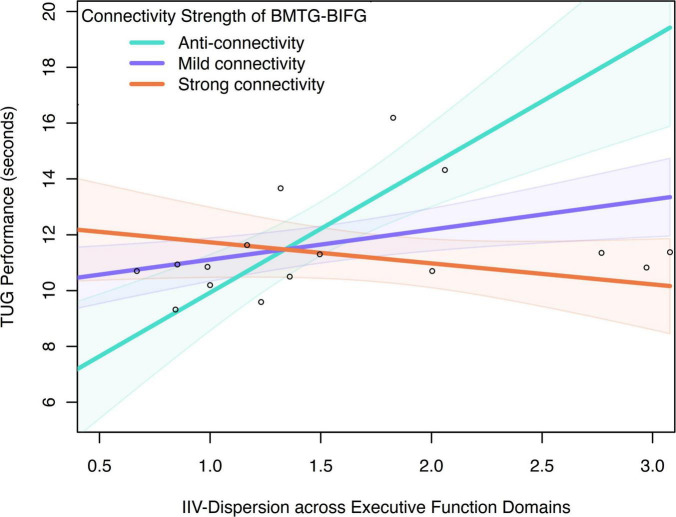
Moderation effect of connectivity between BMTG and BIFG on the association between IIV-dispersion across executive function domains and TUG performance in the CF group.

Moderating relationship between BMTG and BIFG, IIV-dispersion and TUG was only observed in the CF group (R-sq = 0.668, F = 8.706, *p* = 0.002; Table 3). Specifically, IIV-dispersion exerted a significant positive main effect on the TUG performance (β = 2.164, SE = 0.477, *p* < 0.001, 95% CI 7.023, 10.134), indicating that greater IIV-dispersion was associated with longer completion time for TUG test. A significant two-way interaction between IIV-dispersion and functional connectivity of BMTG-BIFG was detected (ΔR^2^ = 0.427, β = −5.719, SE = 1.400, *p* = 0.001, 95% CI [−8.743, −2.695]; Figure 5 and Table 3), accounting for 42.680% of the additional variance in the TUG performance (F = 16.695, *p* = 0.001). Namely, BMTG-BIFG anti-connectivity was conducive toward the positive association between IIV-dispersion and TUG performance (β = 4.082, SE = 0.862, *p* < 0.001, 95% CI [2.219, 5.945]; Figure 5). For those with mild BMTG-BIFG connectivity (i.e., mild connectivity), we observed a significantly weakened positive association between IIV-dispersion and TUG performance (β = 1.561, SE = 0.396, *p* = 0.002, 95% CI [0.705, 2.417]; Figure 5). For those with strong BMTG-BIFG connectivity (i.e., strong connectivity), we observed a notable but non-statistically significant negative relationship between IIV-dispersion and TUG performance (β = −0.960, SE = 0.576, *p* = 0.120, 95% CI [−2.205, 0.285]; Figure 5). Using the Johnson–Neyman technique, we found that the positive association between IIV-dispersion and TUG performance weakened as connectivity increased, suggesting that among community-dwelling older adults with cognitive frailty, those who had greater BMTG-BIFG connectivity may be better protected against balance and mobility impairment even with high levels of IIV-dispersion.

**TABLE 3:** Linear regression model for the CF group.

		β	SE	T	*P*	95% CI	
						LL	UL
**TUG performance**							
Constant		8.579	0.720	11.913	0.000	7.023	10.134
IIV-dispersion		2.164	0.477	4.533	0.001	1.132	3.195
BMTG-BIFG		7.653	2.351	3.255	0.006	2.572	12.733
IIV-dispersion * BMTG-BIFG		−5.719	1.400	−4.086	0.001	−8.744	−2.695
Functional Connectivity	Mean-SD	−0.336	0.862	4.735	0.000	2.219	5.945
	Mean	0.105	0.396	3.941	0.002	0.705	2.417
	Mean+SD	0.546	0.576	−1.667	0.120	−2.205	0.285

CF, cognitively frail older adults; SE, standard error; CI, confidential interval; UL, upper limit; LL, lower limit; TUG, timed-up-and-go test; IIV, intraindividual variability; BMTG, bilateral middle temporal gyrus; BIFG, bilateral inferior frontal gyrus; SD, standard deviation.

No significant moderation effect of functional connectivity of BMTG-BIFG on the associations between IIV-dispersion and TUG performance was observed in the non-CF group (R-sq = 0.013, F = 0.070, *p* = 0.975).

## 4 Discussion

Our cross-sectional study suggests that older adults with cognitive frailty exhibit greater IIV-dispersion and aberrant functional connectivity patterns in the DMN, FEN, and SMN compared to those without cognitive frailty. Furthermore, our findings indicated that the moderating effect of functional connectivity between the DMN-FEN was only notable in individuals with cognitive frailty, not in those without cognitive frailty, such that it may be reflective of an intrinsic adaptive resilience mechanism.

### 4.1 Group differences in IIV-dispersion

Our findings concur with previous studies demonstrating that cognitively impaired older populations showed greater IIV-dispersion than those who were cognitively unimpaired (Halliday et al., 2018; Hilborn et al., 2009). Specifically, a study demonstrated that higher IIV-dispersion across three cognitive domains (i.e., fluid reasoning, executive function, and memory), was associated with increased risk of developing MCI and dementia (Hilborn et al., 2009). Halliday et al. (2018) have also found that older adults with AD had greater IIV-dispersion which was associated with greater impairment in cognitive function across three cognitive domains (i.e., attention, memory, and executive function) compared with cognitively unimpaired older adults or older adults with MCI. Additionally, research examining IIV-dispersion both within and between cognitive domains revealed that IIV-dispersion within the frontal-subcortical domain differentiated older adults with AD from those with normal cognition. In contrast, IIV-dispersion across domains (i.e., language, immediate memory, delayed memory, and frontal-subcortical domains) distinguished older adults with AD not only from those with normal cognition but also from those with MCI (Scott et al., 2023).

Our results confirm and extend these findings to an older population characterized by physical frailty in addition to probable MCI. Specifically, we demonstrated that IIV-dispersion calculated within executive function (i.e., response inhibition and set-shifting) can effectively differentiate older adults with cognitive frailty from those without. These findings imply that IIV-dispersion within and between cognitive domains may have different but complementary roles in identifying and characterizing cognitive decline across varying levels of health status in older adults.

### 4.2 Group differences in functional connectivity

We found that the CF group exhibited notably greater regional DMN-SMN anti-connectivity, as well as lower regional DMN-FEN, DMN-SMN, and within-SMN connectivity, compared to the non-CF group. This may imply a disruption in the network interaction of cognitive and motor processes, potentially underlying cognitive frailty (Wig, 2017). Previous studies suggest that alterations in DMN, FEN, and SMN functional organization were independently associated with cognitive decline and physical frailty in older adults (Voss et al., 2012; Yang et al., 2023; Zhu et al., 2016). For instance, in a cross-sectional study comprised of cognitively unimpaired older adults, older adults with MCI, and older adults with AD, researchers found that compared with those with AD, older adults with MCI exhibited greater anti-connectivity between DMN and the salience network. Likewise, studies illustrated that compared with those with MCI and AD, functional connectivity between the DMN and the salience network was greater in cognitively unimpaired older adults (Zhu et al., 2016). Similarly, cognitively impaired older adults demonstrated decreased connectivity between the DMN and cingulo-opercular network, which contains several overlapping anatomical regions and shares functional involvement in higher-order cognitive processing as the FEN (Voss et al., 2012; Yang et al., 2023). Additionally, compared to non-frail older adults, older adults with physical frailty showed aberrant functional connectivity within SMA network, which includes several overlapping anatomical regions and functionally involved in motor execution and initiation as the SMN (Lammers et al., 2020).

Our results extend current knowledge by illustrating the differences in connectivity patterns in cognitively frail individuals. The significantly lower intra-network connectivity of task-oriented network (i.e., SMN) relative to the non-CF group highlighted the loss of regional modularity, which may be indicative of disrupted local network efficiency due to cognitive frailty. Further, we found that compared with the CF group, the non-CF group had significantly weaker DMN-related anti-connectivity with select large-scale networks. Loss of functional specialization (i.e., dedifferentiation) is considered part of the aging process (Rodriguez-Sabate et al., 2019). However, the Scaffolding Theory of Aging and Cognition posited that compensatory scaffolding is an essential component of healthy aging (Park and Reuter-Lorenz, 2009), such that the recruitment of additional neural circuits may be represented by increased connectivity between functional networks. Therefore, it is possible that the notable anti-connectivity between DMN-FEN, and DMN-SMN observed in the CF group were not reflective of a brain state similar to those of younger adults, rather it is likely that the network configurations we observed in older adults with cognitive frailty may be reflective of the inability to form successful compensatory inter-network connections to account for the compromised neural integrity and functional capacity. Future longitudinal studies will be necessary to confirm this proposition.

Previous studies suggested that greater IIV-dispersion was associated with lower intra-network connectivity of the DMN (Lin and McDonough, 2022; Mulet-Pons et al., 2023a). Contrary to these findings, we found no statistically significant group differences within the DMN. This discrepancy may be rooted in the methodology and population used in previous research, which primarily focused on the relationship between IIV-dispersion and connectivity within the DMN in cognitively unimpaired middle and older individuals. In contrast, there has been limited examinations of the differences in IIV-dispersion and within DMN connectivity among older adults with cognitive frailty. Therefore, findings from this study extends previous evidence and provide preliminary insights into intrinsic shifts in DMN connectivity paradigm (i.e., intra- vs. inter-network) that may be specific to cognitive frailty.

Alternatively, it is important to point out that compared with the CF group, the non-CF group had greater (trend-level; *p* = 0.06) years of education–a well-established proxy of cognitive reserve. Therefore, the lack of observable differences in intra-network DMN connectivity between older adults with and without cognitive frailty in this study may be reflective of a scenario where, despite similar levels of DMN deterioration, neural protective effects of greater cognitive reserve actively supported the maintenance of cognitive and physical function. Given these observations, we further posit that intra-network connectivity of the DMN is plausibly segregated from neural architecture underpinning cognitive reserve. Conversely, connectivity between the DMN and other functionally recruited brain regions may be more aligned with the adaptive properties of reserve-related brain reorganization. Subsequent neuroimaging studies with greater sample size will be required to rigorously test our assumption.

Interestingly, we found no significant group difference in the inter-network connectivity of FEN and SMN. This lack of difference indicates that FEN-SMN connectivity may be less susceptible to impacts of cognitive frailty. Studies showed that older adults with cognitive impairments primarily experience disruptions in the connectivity of the DMN with other major networks (Yang et al., 2023; Zhu et al., 2016). While studies on physically frail older adults have reported attenuated connectivity primarily in cortical areas associated with motor function (i.e., SMN) (Lammers et al., 2020), neural correlates of additional cognitive burden on top of frailty remains unclear. Our findings suggest that internetwork connection to the FEN may not be affected by cognitive frailty albeit the FEN’s conventional association with high order cognitive processes (Dosenbach et al., 2006). Instead, connectivity to the DMN may be more sensitive to aging-related cognitive-motor comorbidity, which aligns with the established role of the DMN in cognitive processing (Wang et al., 2017). For instance, regions of the DMN are involved in sensory motor processing, and executive functions (Wang et al., 2017). Additionally, Hsu et al. (2020) found that aberrant connectivity of DMN-SMN was associated with worse mobility performance in older adults with MCI. This is further supported by our findings of significant differences in the inter-network connectivity of DMN-FEN and DMN-SMN between older adults with and without cognitive frailty.

Behaviorally, we observed significantly shorter sleep duration and lower physical activity level in the CF group compared with the non-CF group. Research indicates that shorter sleep duration and decreased physical activity negatively impact various cognitive function domains (i.e., attention and working memory) in older adults (Falck et al., 2017). However, we did not observe significant associations between IIV-dispersion, sleep duration, and PASE (see Supplementary Table 5 for full details). The lack of a relationship observed in our study may be due to subjective recall of total sleep duration and physical activity not adequately capturing their complexity or variability. For instance, a previous study found a significant association between sleep variability (i.e., night-to-night variation in sleep duration and quality) and IIV-inconsistency in older adults with cognitive impairments (Balouch et al., 2022). Additionally, another study indicated that IIV-inconsistency was negatively associated with daily duration of moderate physical activity over three months measured by an electronic accelerometer in older adults (Kimura et al., 2013). This implies that objectively measurements of sleep variability and physical activity may be more closely linked to IIV-dispersion.

### 4.3 Moderation effect of connectivity of DMN-FEN on the association between IIV-dispersion and TUG in the CF group

Our study demonstrated that among older individuals with cognitive frailty, DMN-FEN connectivity was associated with preserved TUG performance in face of higher IIV-dispersion. Greater internetwork DMN connectivity observed may represent an adaptive intrinsic resilience mechanism that enables certain cognitively frail individuals to flexibly recruit neural resources, thereby supporting the maintenance of TUG performance even under high levels of IIV-dispersion, a notion that may be linked to the concepts of physical reserve (Holtzer et al., 2023).

Physical reserve (PR), as conceptualized by O’Brien and Holtzer (2023), refers to an individual’s capacity to maintain physical functioning in the face of aging, illness, or injury (O’Brien and Holtzer, 2023). This emerging construct of resilience emphasizes the brain’s ability to efficiently or compensatorily reallocate neural resources through functional brain networks, thereby sustaining physical performance despite age- or disease-related neural changes (Holtzer et al., 2023). For example, Hsu et al. (2024) found that cognitively impaired older adults with higher physical reserve–underpinned by internetwork connectivity of the frontal-parietal network and the SMN, displayed better posture stability in face of extensive white matter lesion than their counterparts with lower physical reserve. These studies suggest that adaptability of brain networks may enable older adults to mitigate impaired cognitive function and mobility, which is highly relevant to cognitive frailty. Additionally, neural imaging study has highlighted the role of the FEN in motor plasticity (Newbold et al., 2021). Thus, our findings suggest that DMN-FEN connectivity may serve as a neural substrate for resilience among cognitively frail older individuals for maintaining TUG performance even in the presence of cognitive dysfunction (i.e., high levels of cognitive intraindividual variability). Future longitudinal studies with larger sample sizes and lesion quantification will be necessary to confirm the neural protective properties of physical reserve in mitigating TUG performance decline associated with high IIV as a consequence of cognitive frailty. In the non-CF group, no significant moderation effect of connectivity of DMN-FEN on the association between IIV and TUG performance was observed. It is probable that these individuals did not require adaptive reorganization of functional networks given the unimpaired cognitive and physical capacity.

### 4.4 Strength and limitations

The primary strength of our study lies in its novel uncovering of the association between IIV, TUG performance, and brain function. This finding may help refine current intervention strategies aimed at reducing impairments in TUG performance associated with high IIV resulting from cognitive frailty. However, several limitations should be discussed. We included only cognitively frail older adults who were independent enough to participate in research studies and MRI, which may limit the generalizability of our findings. PASE is a subjective assessment tool that is vulnerable to recall bias in participants, particularly among older adults, which may compromise the reliability of the data. FCI is a tool based on medical diagnoses instead of symptoms, therefore the cumulative scores may not accurately capture the full spectrum of current or evolving comorbidities if the participants did not undergo recent medical examinations. Additionally, the relatively small sample size restricted the statistical power of our analyses and may have reduced the robustness and generalizability of the observed associations. Caution is needed when interpreting our results. The limited number of cognitive tests used to compute IIV-dispersion in this study highlights the potential value of incorporating a broader range of cognitive domains in future research to derive a more robust estimate of IIV-dispersion. Furthermore, longitudinal studies are necessary to fully understand the compensatory mechanisms implicated by physical reserve (or other resilience mechanisms) that support the maintenance of cognition and physical function in the cognitively frail population. Moreover, future studies including additional groups (i.e., individuals with probable MCI only and those with physical frailty only) are needed to elucidate the independent and interactive effects of IIV-dispersion and brain functional connectivity in differentiating cognitive frailty from probable MCI, physical frailty, and non-cognitive frailty.

### 4.5 Potential clinical implication

Results from this study will offer insights on how increased IIV-dispersion in individuals with cognitive frailty may be an important clinical indicator for mobility impairment. We demonstrated that evaluation of IIV-dispersion can complement existing methods to better identify individuals with cognitive frailty who are at risk of mobility impairment, enabling the timely preventative strategies and interventions. To promote functional recovery in this population, integrating interventions that simultaneously address the specific cognitive (i.e., IIV-dispersion) and mobility (i.e., TUG) challenges experienced by people with cognitive frailty will be critical. By elucidating the functional neural mechanism, we found that connectivity between DMN and FEN may be the central neural correlate of cognitive and mobility decline. Older adults with CF may benefit from targeted treatments that focus specifically on enhancing inter-network connectivity between the DMN and FEN. Collectively, these findings suggest that current treatment may be refined by incorporating neuromodulatory methods, such as physical exercise and brain stimulation. For instance, Voss et al. (2010) demonstrated that a 12-month aerobic training program enhanced DMN-FEN connectivity that was correlated with improved executive function in older adults (Voss et al., 2010). Similarly, single-pulse transcranial magnetic stimulation induced changes in connectivity between the DMN and cognitive network that enhanced cognitive processing under load (Webler et al., 2022).

## 5 Conclusion

This cross-sectional study provided preliminary evidence for the relationship between IIV-dispersion, TUG performance, and functional network connectivity in older adults with and without cognitive frailty. Our findings indicated that adaptive reorganization of DMN-FEN connectivity may be part of the underpinning compensatory neural mechanism associated with preserved TUG performance despite impaired cognitive function. These insights could help establish sensitive screening tool for identifying individuals with cognitive frailty, as well as help refine intervention strategies to prevent further mobility impairment in older adults experiencing concurrent cognitive and physical challenges.

## Data Availability

The datasets presented in this study can be found in online repositories. The names of the repository/repositories and accession number(s) can be obtained by contacting the corresponding author.
