# Supplementary material for: Brain connectivity moderated the effects of cognitive intraindividual variability on mobility in cognitively frail older adults
Source: Front Aging Neurosci. 2025 Oct 31;17:1682996. doi: 10.3389/fnagi.2025.1682996 (PMC12615459; doi:10.3389/fnagi.2025.1682996)
Supplement: Supplementary file 1 [file Data_Sheet_1.docx]

Supplementary Material

Brain connectivity moderated the effects of cognitive intraindividual variability on mobility in cognitively frail older adults

Jingyi Wu, MSc^1^; Jinyu Chen, MSc^1^; Juncen Wu, MSc^1^; Wayne Lap Sun Chan, PhD^1^; Yijian Yang, PhD^2^; Chun Liang Hsu, PhD^1*^

1. Department of Rehabilitation Sciences, Faculty of Health and Social Sciences, The Hong Kong Polytechnic University, Hong Kong SAR, China
2. Department of Sports Science and Physical Education, The Chinese University of Hong Kong, Hong Kong SAR, China

* Corresponding author:

Chun Liang Hsu, PhD,

Department of Rehabilitation Sciences,

Faculty of Health and Social Sciences,

The Hong Kong Polytechnic University,

# 11 Yuk Choi Rd, Hung Hom,

Hong Kong SAR, China

Phone: +852 27666755

1. mail: [chun-liang.hsu@polyu.edu.hk](mailto:chun-liang.hsu@polyu.edu.hk)

ORCID: 0000-0003-2090-1001

**Online Supplementary Material**

| Table S1. Neural Networks and Regions of Interests Included in the Analysis | Page 2 |
| --- | --- |
| Figure S1. Linearity Plot of Moderation Model in the CF group | Page 3 |
| Table S2. Variance Inflation Factors for IIV-dispersion, BMTG-BIFG, and IIV-dispersion: BMTG-BIFG in the CF group | Page 3 |
| Table S3. Linear regression model for the CF group using mean centered IIV-dispersion and BMTG-BIFG | Page 3 |
| Figure S2. Moderation Model | Page 4 |
| Table S4. Participant executive functions, mobility, and network functional connectivity | Page 5 |
| Table S5. Correlation analysis in the CF group | Page 6 |

**Table S1.** Neural Networks and Regions of Interests Included in the Analysis

| Neural Networks | Regions of Interests | MNI Coordinates (mm) | | |
| --- | --- | --- | --- | --- |
|  |  | x | y | z |
| DMN | PCC | 8 | -56 | 30 |
|  | FMC | -2 | 54 | -12 |
|  | LMTG | -52 | -14 | -20 |
|  | RMTG | 58 | -10 | -18 |
|  | LPHG | -26 | -24 | -20 |
|  | RPHG | 24 | -26 | -20 |
|  | LMFG | -30 | 20 | 50 |
|  | LLOC | -44 | -72 | 30 |
|  | RLOC | 54 | -62 | 32 |
| FEN | RALPFC | 32 | 40 | 28 |
|  | LINS | -38 | 8 | -4 |
|  | RINS | 38 | 4 | -2 |
|  | LPFC | -36 | 34 | 28 |
|  | RPFC | 32 | 42 | 36 |
|  | LIFG | -38 | 48 | 8 |
|  | RIFG | 34 | 48 | -6 |
|  | CING | 4 | 28 | 26 |
| SMN | LPCG | -39 | -21 | 55 |
|  | RPCG | 34 | -25 | 53 |
|  | LCB | -24 | -66 | -19 |
|  | RCB | 25 | -71 | -23 |
|  | LPM | -16 | 0 | 57 |
|  | RPM | 20 | -17 | 61 |
|  | SMA | -5 | -1 | 52 |

DMN: default mode network; FEN: fronto-executive network; SMN: sensorimotor network; BIFG: bilateral inferior frontal gyrus; SMA: supplementary motor area; DMN: default mode network; BMTG: bilateral middle temporal gyrus; RALPFC: right anterior lateral prefrontal cortex; LMFG: left middle frontal gyrus; BLOC: bilateral lateral parietal cortex; LCB: left cerebellum; LPCG: left precentral gyrus; RCB: right cerebellum; RPCG: right precentral gyrus; FMC: frontal medial cortex.


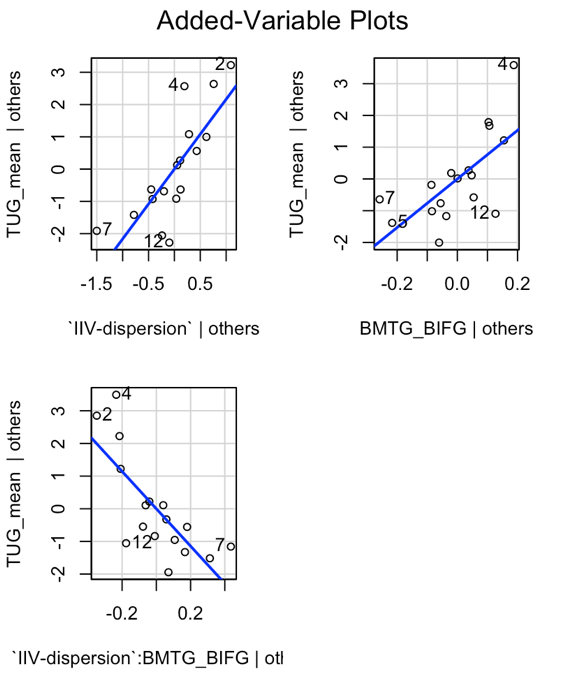


Figure S1. Linearity plots of moderation model in the CF group

Table S2. Variance Inflation Factors for IIV-dispersion, BIFG-BMTG, and IIV-dispersion:BMTG-BIFG

| Variable | VIF |
| --- | --- |
| IIV-dispersion_c | 1.273 |
| BMTG-BIFG_c | 1.082 |
| IIV-dispersion_c:BMTG-BIFG_c | 1.298 |

CF: cognitively frail older adults; c: mean center

Table S3. Linear regression model for the CF group using mean centered IIV-dispersion and BMTG-BIFG

|  | | **β** | **se** | **t** | ***P*** | **95%CI** | |
| --- | --- | --- | --- | --- | --- | --- | --- |
|  |  |  |  |  |  | **LL** | **UL** |
| TUG performance | |  |  |  |  |  |  |
| constant | | 11.762 | 0.297 | 39.561 | 0.000 | 11.120 | 12.404 |
| IIV-dispersion_c | | 1.561 | 0.396 | 3.941 | 0.002 | 0.705 | 2.417 |
| BMTG-BIFG_c | | -1.057 | 0.680 | -1.554 | 0.144 | -2.527 | 0.413 |
| IIV-dispersion_c * BMTG-BIFG_c | | -5.719 | 1.400 | -4.086 | 0.001 | -8.744 | -2.695 |
| Functional Connectivity | Mean-SD | -0.522 | 0.967 | 4.703 | 0.000 | 2.459 | 6.637 |
|  | Mean | 0.087 | 0.362 | 2.942 | 0.012 | 0.283 | 1.848 |
|  | Mean+SD | 0.412 | 0.545 | -1.457 | 0.169 | -1.972 | 0.384 |

CF: cognitively frail older adults; se: standard error; CI: confidential interval; LL: lower limit; UL: upper limit; TUG: timed-up-and-go test; IIV: intraindividual variability; BMTG: bilateral middle temporal gyrus; BIFG: bilateral inferior frontal gyrus; c: mean center; SD: standard deviation


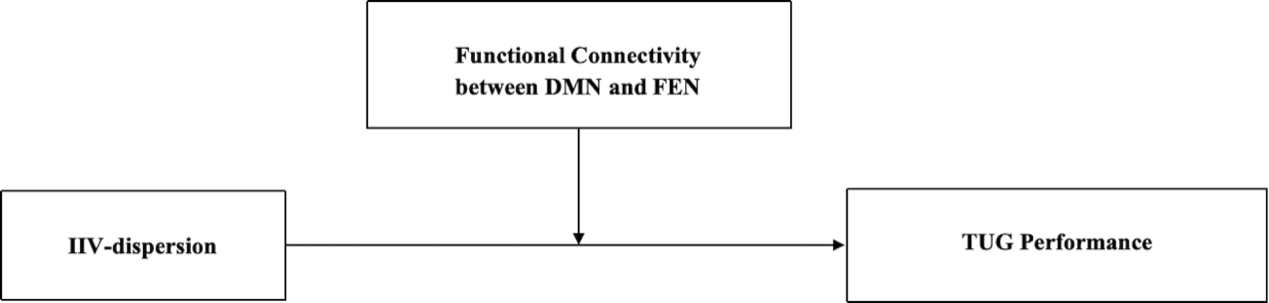


**Figure S2.** Moderation Model

Table S4. Participant executive functions, mobility, and network functional connectivity

| **Variables** | **Mean (SD)** | | **Adjusted Mean (SE)** | | **Effect Size (η²)** | **P Value** |
| --- | --- | --- | --- | --- | --- | --- |
|  | **CF group**  **(n=17)** | **Non-CF group (n=20)** | **CF group**  **(n=17)** | **Non-CF group (n=20)** |  |  |
| **Mobility Measure** | | | | | |  |
| TUG | 11.349 (1.802) | 8.216 (1.545) | 11.205 (0.436) | 8.338 (0.398) | 0.388 | 0.000 |
| **Executive Function Measure** | | | | | | |
| Trail Making Test A | 45.404 (20.578) | 32.324 (9.724) | 42.849(3.688) | 34.495 (3.364) | 0.070 | 0.125 |
| Trail Making Test B | 152.341 (75.390) | 80.977 (24.643) | 142.842 (11.556) | 89.051 (11.541) | 0.241 | 0.003 |
| Trail Making Test  B-A | 106.936 (65.619) | 48.654 (19.199) | 99.992 (10.508) | 54.556 (9.585) | 0.215 | 0.005 |
| Stroop I | 49.145 (10.887) | 40.801 (6.225) | 47.683 (2.079) | 42.043 (1.897) | 0.097 | 0.068 |
| Stroop II | 63.905 (14.711) | 52.753 (7.420) | 62.763 (2.985) | 53.723 (2.723) | 0.118 | 0.043 |
| Stroop III | 122.764 (30.514) | 95.014 (21.641) | 123.288 (7.007) | 94.569 (6.391) | 0.197 | 0.008 |
| Stroop interference | 58.858 (24.256) | 42.262 (18.087) | 60.524 (5.642) | 40.845 (5.146) | 0.151 | 0.021 |
| IIV-dispersion | 1.523 (0.821) | 0.943 (0.459) | 1.403 (0.166) | 1.045 (0.510) | 0.063 | 0.145 |

Covariate was evaluated at FCI = 1.30, years of education=12.11

SD: standard deviation; SE: standard error; CF: cognitively frail older adults; TUG: Timed-up-and-go test

Table S5. Correlation between IIV-dispersion and participant characteristics in the CF group

| Variable | Sleep duration over 24h | Sleep duration over 7 days | PASE | Years of Education |
| --- | --- | --- | --- | --- |
| IIV-dispersion | -0.258 | -0.172 | -0.475 | -0.319 |

CF: cognitively frail older adults; PASE: Physical Activity Scale for the Elderly
